# Micturition in the toilet compared with bedpan in laboring Nulliparas: a randomized controlled trial

**DOI:** 10.1186/s12884-022-05162-4

**Published:** 2022-11-04

**Authors:** Maherah Kamarudin, Wen Kiat Chong, Mukhri Hamdan, Aizura Syafinaz Adlan, Rahmah Saaid, Peng Chiong Tan

**Affiliations:** Department of Obstetrics & Gynecology, Faculty of Medicine, Universiti Malaya, Lembah Pantai, 50603 Kuala Lumpur, Malaysia

**Keywords:** Nulliparas, Labor, Toilet, Bedpan, Micturition, Urination, Bladder voiding, Randomized trial

## Abstract

**Background:**

Bladder overdistension in labor may lead to prolonged postpartum urinary retention. We hypothesized that nulliparas mobilizing to toilet is more likely to achieve satisfactory micturition.

**Methods:**

One hundred sixteen (58 in each arm) term nulliparas in labor with filled bladders were randomized to mobilizing to the toilet or using bedpan to micturate. Primary outcome was satisfactory micturition defined as ultrasound derived post-void bladder volume < 150 ml. Following unsatisfactory micturition, participants crossover to the opposed intervention. Participants were catheterized if after crossover, residual bladder volume was ≥250 ml.

**Results:**

Satisfactory micturition rates were 55/58 (95%) vs. 43/58 (74%) RR 1.28 95%CI 1.08–1.51 NNT_b_ 4.8 95%CI 3.0–12.4 *P* = 0.008, failure to micturate 1/58 (2%) vs. 8/58 (14%) RR 0.13 95%CI 0.02–0.97 NNT_b_ 8.3 95%CI 4.6–38.7 *P* = 0.047. After cross over following unsatisfactory bladder voiding, satisfactory micturition rates were 0/3 (0%) vs 13/15 (87%) *P* = 0.024, bladder catheterization rates were 3/58 (5%) vs. 2/58 (4%) RR 95%CI 1.5 (0.26–8.65) *P* = 0.648, maternal satisfaction with allocated intervention 55/58 (95%) vs. 9/58 (16%) RR 95%CI 6.1 (3.3–11.2) NNT_b_ 95%CI 1.3 (1.1–1.5) *P* <  0.0001 and preference for mobilizing to the toilet if micturition was needed again during labor 55/58 (95%) vs. 53/58 (92%) for mobilizing to the toilet compared to bedpan use arms respectively. Labor and neonatal outcomes were similar.

**Conclusion:**

Satisfactory micturition was more frequently achieved with mobilization to the toilet than bedpan use. Women in both arms overwhelmingly prefer to mobilize to the toilet to urinate.

**Trial registration:**

This study was registered with ISRCTN on 17/07/2019 with trial identification number: ISRCTN17787339. First participant was recruited on 31/07/2019. The last patient was recruited on 18/12/2019.

**Supplementary Information:**

The online version contains supplementary material available at 10.1186/s12884-022-05162-4.

## Background

Overstretching the bladder wall during pregnancy or delivery can result in severe detrusor damage followed by voiding dysfunction [1]. Acute covert postpartum urinary retention rate is reported at 10.6% [2], overt retention at 0.3% [2] and prolonged retention persisting for over 12 months at 0.07% [3]. First labors last on average 8 hours and birth expected within 3 hours of pushing whereas subsequent labors last on average 5 hours and birth expected within 2 hours of pushing [4]. Nulliparity and prolonged labor particularly in the second stage are well-established risk factors for postpartum voiding dysfunction [5, 6].

Nulliparas due to their tendency for labor dystocia, often need oxytocin augmentation [7, 8] with concomitant continuous cardiotocography, intravenous hydration [9] and neuraxial analgesia as their labor pain is perceived to be more intense [10]; hence are often connected to multiple infusion and monitoring devices limiting mobility during labor. Care providers maybe wary of interrupting monitoring and treatment, and of inadvertent birth into the toilet when mobilizing laboring women to micturate in the toilet. In our busy publicly funded university hospital, bedpan use or even catheterization is increasingly seen as time and labor-saving for intrapartum bladder care. The UK National Institute of Clinical Excellence (NICE) guidance recommends recording frequency of passing urine during first stage of labor without further elaboration on bladder voiding management.

Women’s toileting behavior related to micturition can be defined as voluntary actions related to the physiological event of emptying the bladder comprising specific attributes of voiding place, voiding time, voiding position and voiding style influenced also by the physical and social environments [11]. For these reasons, it is postulated that micturition in the toilet is more likely to be satisfactorily performed compared to with bedpan use for nulliparas at high risk of in-labor voiding dysfunction. We sought to evaluate this hypothesis in a randomized trial.

## Methods

This trial was approved by the Medical Ethics Committee of University Malaya Medical Centre (reference number: 2019330–7272 on 04/07/2019) and registered with ISRCTN (https://doi.org/10.1186/ISRCTN17787339 on 17/07/2019). The trial was performed in accordance with International Conference on Harmonization – Guidelines for Good Clinical Practice (ICH-GCP) and Declaration of Helsinki. Informed consents were obtained from all participants.

We sought in our labor ward, nulliparas (no prior delivery beyond 20 weeks gestation) in labor (at least three contractions in 10 minutes, cervical dilatation within the last 2 hours of 4 to 8 cm and ruptured membrane but without evidence of second stage) who had the urge to micturate or a palpable bladder and ultrasound scan bladder volume > 300 ml, aged > 18 years old with a singleton live fetus in cephalic presentation for trial participation. An adult functional bladder can comfortably hold between 300 and 400 ml [12]. Wall pressure of 5–15 mmHg creates a sensation of bladder fullness while 30 mmHg and beyond is painful [13]. For this trial a filled bladder that warrants voiding is defined as the urge to micturate or a palpable bladder and ultrasound bladder volume > 300 ml [14]. Women with known bladder dysfunction, recurrent antenatal urinary tract infections, lower segment uterine fibroid, on neuraxial analgesia and prior bedpan or urine catheter use during labor were excluded. Eligible women were approached, verbally informed and provided with the participant information sheet by the investigator (WKC). Written informed consents were obtained from all participants. Relevant demographic and clinical data were recorded in the Case Report Form.

Pre and postvoid ultrasound for bladder volume was performed (solely by investigator WKC) using a 3.5 MHz convex transducer with the participant in semi-recumbent position. Bladder measurements were performed in two planes (transverse and sagittal images) in between contractions. For transverse image, the transducer is positioned horizontally along the long axis of the bladder, and adjusted until the maximum transverse section is found and the maximum transverse width measured (horizontal diameter). For the sagittal image, the transducer is rotated 90-degree into the sagittal plane, position adjusted until the maximum sagittal section is obtained and sagittal depth and height were then measured. Sonographic measurements were recorded for sagittal height, sagittal depth and transverse width of the bladder (i.e., h, d and w). Using the prolate ellipsoid method [15], volume = 0.52 x h x d x w [16].

Prior to randomization women were asked on their preferred method to urinate using bedpan, mobilize to toilet, catheterization or no preference. Participants were randomized to micturition by mobilizing to toilet or using bedpan by opening the lowest numbered, sealed and opaque envelope that remained. The randomization sequence was computer generated in blocks of 4 or 8 using random.org by investigator (PCT) who was not involved with recruitment.

Participants randomized to mobilizing to toilet were disconnected from medical devices and can choose to go to the toilet with a companion (birth partner or the midwife) or by themselves with the door closed, for up to 10 min to micturate on a standard sitting toilet. The voided urine was collected into a disposable plastic bag that was hold firmly under the toilet lid. The voided urine volume was measured after decanting to a measuring jug. Participants in the bedpan arm was provided with the bedpan and be in any position that was comfortable to her (lying or sitting on the bed) for up to 10 min to micturate. The voided volume was measured after decanting to a measuring jug.

Primary outcome of satisfactory micturition is defined as residual urine volume < 150 ml by ultrasound. A residual bladder volume ≥ 150 ml typically defines covert postpartum urinary retention [17, 18]. Participants who were not able to micturate, or if their residual urine volume ≥ 150 ml, were asked to cross-over to the opposite intervention for another attempt. If after attempting both methods and residual urine volume was > 250 ml, a standard “in and out” bladder catheterization was offered and performed under an aseptic technique.

Immediately after completion of their micturition attempt, participants provided a 5-grade Likert’s scale response of their satisfaction with their allocated intervention and stated their preferred bladder voiding method to “if you have to pass urine again during labor, what would you prefer?”. Labor and neonatal outcomes were retrieved from participants’ charts. Prespecified secondary outcomes were in-out catheterization, duration of the second stage of labor, mode of delivery, estimated delivery blood loss and maternal satisfaction after allocated intervention prior to any crossover.

Participants, investigator (WKC) who performed the ultrasound and care providers were not masked to the interventions as it was impractical due to its obvious nature.

As there was no direct pilot data available, sample size calculation was based on in-out catheterization rates of 19% vs 54% in recovery room among postoperative patients (early mobilization vs bedpan) [19] as surrogate outcome to represent unsatisfactory micturition of this study. We applied a more conservative difference of 25% vs 50%, alpha 0.05, power 80%, 1:1 randomization and Chi-square test, 58 participants were needed in each arm (*N* = 116) using the PS: Power and Sample Size Calculation [20].

Data were entered into SPSS (Version 23, IBM, SPSS Statistics). Student t test was applied for means with normally distributed continuous data, Mann-Whitney U test for non-normally distributed data or ordinal data, Chi-square test for categorical data across trial arms and paired t test for continuous data within trial arms. Two-sided *P* values were reported and *p* <  0.05 was regarded as significant. Analysis was based on intention-to-treat.

## Results

**Fig. 1:**
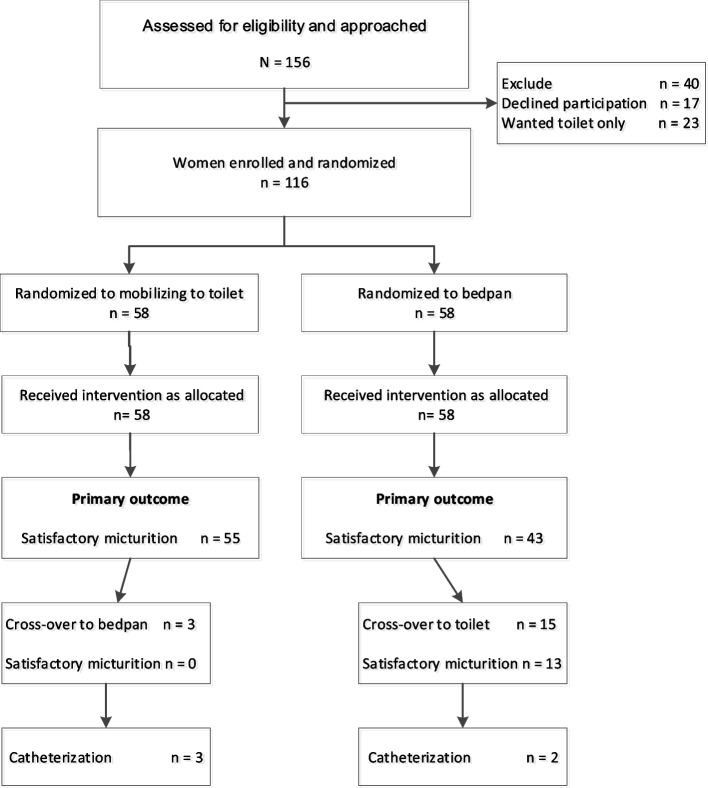
Recruitment flow chart into a randomized trial of mobilization to the toilet compared with bedpan use to micturate in nulliparas with a filled bladder during labor

Figure 1 depicts the recruitment flow: 156 potentially eligible women were approached, 17 declined participation, and 23 women refused to be randomized, wanting mobilization to the toilet. Recruitment stopped on reaching target sample size of 116 (58 randomized to each arm).

Table 1 shows trial participants’ characteristics: prevoid bladder volume, contraction frequency, cervical dilatation and bladder voiding preferences in particular were similar across trial arms.

**Table 1:** Characteristics of trial participants randomized to mobilization to the toilet compared with bedpan use to micturate in nulliparas with a filled bladder during labor

Characteristics	Mobilizing to toilet *n* = 58	Bedpan *n* = 58	*P* value
Age (years)	29.6 ± 3.3	28.7 ± 4.8	0.242
Body mass index (kg/m^2^)	28.6 ± 4.8	29.0 ± 4.8	0.717
Gestational age at recruitment (weeks)	39.1 ± 1.2	39.2 ± 1.0	0.661
Ethnicity			
Malay	33 (57)	34 (59)	0.635
Chinese	10 (17)	10 (17)	
Indian	10 (17)	6 (10)	
Others^a^	5 (9)	8 (7)	
Education level			0.402
Up to primary	0 (0)	2 (3)	
Secondary	10 (17)	11 (19)	
Diploma	17 (29)	20 (35)	
Degree	31 (53)	25 (43)	
Occupation			
Paid employment	48 (83)	43 (74)	0.367
Housewife and students	10 (17)	15 (26)	
Pre-void bladder volume (ml)	444 ± 104	441 ± 93	0.655
Contraction intensity			
3 in 10 minutes	37 (64)	33 (57)	0.569
≥ 4 in 10 minutes	21 (36)	25 (43)	
Cervical dilatation			
< 6 cm	48 (83)	49 (85)	> 0.99
≥ 6 cm	10 (17)	9 (15)	
Pre-randomization preference			0.798
Mobilize to toilet	50 (86)	48 (83)	
Bedpan	0 (0)	0 (0)	
No preference	8 (14)	10 (17)	
Catheterization	0 (0)	0 (0)	

Data expressed as mean ± standard deviation or number (%). Analyses by Student t test for comparison of means for continuous data and Chi-Square test categorical datasets. 2-sided analyses

^a^ other ethnicities: 4 Indonesian, 4 Myanmar national, 2 Sabah native, 2 Filipino, 1 Yemeni

Table 2 reports the primary outcome and other micturition related outcomes. Primary outcome of satisfactory micturition was 55/58 (95%) vs. 43/58 (74%) RR 95%CI 1.28 (1.08–1.51) NNT_b_ 95%CI 4.8 (3.0–12.4) *P* = 0.008 and prespecified secondary outcome bladder catheterization 3/58 (5%) vs. 2/58 (4%) RR 95%CI 1.5 (0.26–8.65) *P* = 0.648 for toilet vs. bedpan respectively. Other (post hoc) significant secondary outcomes of failure to micturate (0 ml voided) were 1/58 (2%) vs. 8/58 (14%) RR 95%CI 0.13 (0.02–0.97) NNT_b_ 95%CI 8.3 (4.6–38.7) *P* = 0.047, and after cross-over, successful micturition was 0/3 (0%) vs. 13/15 (87%) *P* = 0.024 for toilet vs. bedpan respectively.

**Table 2:** Primary outcome and other bladder voiding outcomes after randomization to mobilization to the toilet compared with bedpan use to micturate in nulliparas with a filled bladder during labor

Outcomes	Mobilizing to toilet (*n* = 58)	Bedpan (*n* = 58)	RR (95% CI)	NNT_b_ (95% CI)	*P* value
Satisfactory micturition^a^	55 (95)	43 (74)	1.28 (1.08–1.51)	4.8 (3.0–12.4)	0.008
No urine voided	1 (2)	8 (14)	0.13 (0.02–0.97)	8.3 (4.6–38.7)	0.047
Voided volume (measured^b^ – ml)	390 ± 143	321 ± 215			0.374
Post-void volume (ultrasound^c^ - ml)	37.9 ± 97.4	119 ± 183			0.047
Satisfactory micturition^a^	*n* = 55	*n* = 43			
Voided volume (measured^b^ – ml)	410 ± 114	432 ± 120			0.369
Unsatisfactory micturition^d^	*n* = 3	*n* = 15			
Voided volume (measured^b^ – ml)	7 ± 8	4 ± 5			0.523
Satisfactory micturition^a^ after cross-over	0 (0)	13 (87)			0.024
Catheterization after failed^d^ cross-over	3 (5)	2 (4)	1.5 (0.26–8.65)		0.648

Data expressed as number (%) or mean ± standard deviation. Analyses by Student t test for continuous data or Chi Square test for 2 × 2 categorical datasets

^a^Satisfactory micturition is defined as residual bladder volume by ultrasound scan < 150 ml

^b^Voided urine retrieved from receptacle and measured with a calibrated volume measurement jug

^c^Ultrasound formula for bladder content: volume = 0.52 x h (height) x d (depth) x w (width)

^d^Unsatisfactory micturition (failed micturition attempt) is defined as residual bladder volume by ultrasound scan ≥150 ml

Table 3 shows maternal and neonatal secondary outcomes. Prespecified secondary outcomes of maternal satisfaction with allocated intervention was 55/58 (95%) vs. 9/58 (16%) RR 95%CI 6.1 (3.3–11.2) NNT_b_ 95%CI 1.3 (1.1–1.5) *P* < 0.0001, heavily favored mobilization to the toilet arm, but duration of the second stage of labor, mode of delivery and estimated delivery blood loss were not different. When asked to state a preference if they need to micturate in labor again, 55/58 (95%) mobilizing arm vs. 53/58 (92%) bedpan arm *P* = 0.554 responded overwhelmingly and uniformly in favor of mobilization to the toilet in both trial arms. Neonatal outcomes were similar.

**Table 3:** Secondary outcome after randomization to mobilization to the toilet compared with bedpan use to micturate in nulliparas with a filled bladder during labor

Outcomes	Mobilizing to toilet (*n* = 58)	Bedpan (*n* = 58)	RR (95% CI)	NNT_b_ (95% CI)	*P* value
**Maternal outcomes**					
	*n* = 36	*n* = 43			
Duration of second stage (minutes)	22 [14–35]	22 [16–33]			0.953
Mode of delivery					0.258
Spontaneous vaginal	29 (50)	31 (53)			
Instrumental delivery	7 (12)	12 (21)			
Cesarean section	22 (38)	15 (26)			
Estimated blood loss	325 [300–500]	300 [300–400]			0.356
Satisfied with allocated intervention					
Agree^a^	55 (95)	9 (16)	6.1 (3.3-11.2)	1.3 (1.1-1.5)	< 0.0001
Do not agree^a^	3 (5)	49 (85)			
Preference if were to micturate again during labor					0.554
Mobilize to toilet	55 (95)	53 (92)			
No preference	3 (5)	4 (7)			
Bedpan	0 (0)	0 (0)			
Catheterization	0 (0)	1 (2)			
**Neonatal outcome**					
Birth weight (kg)	3.1 ± 0.4	3.1 ± 0.3			0.292
Apgar score (1 min)	9 [9–9]	9 [9–9]			0.721
Apgar score (5 min)	10 [10–10]	10 [10–10]			0.660
Cord arterial blood pH	7.28 ± 0.08	7.29 ± 0.08			0.290
**Neonatal outcome**					
Neonatal admission	5 (9)	2 (3)	0.67 (0.1–3.8)		0.435
Indication for admission					0.646
Transient tachypnea of newborn	1 (20)	1 (50)			
Presumed sepsis	3 (60)	1 (50)			
Subaponeurotic hemorrhage	1 (20)	0 (0)			

Data expressed as number (%), mean ± standard deviation for normally distributed data or median [interquartile range] for non-normally distributed data. Analyses by, Fisher’s exact test for 2 × 2 categorical datasets. Chi square test for larger than 2 × 2 categorical datasets and Mann-Whitney U test for non-parametric data (assessed by Kolmogorov-Smirnov test) or ordinal 2-sided analyses *P* < 0.05 for all variables

^a^Recategorization of Likert’s scale responses, “agree” includes strongly agree or agree, “do not agree” includes neither agree or disagree, disagree or strongly disagree

### Post-hoc analysis

We sought to evaluate a) difference in ultrasound derived mean voided volume (calculated from pre and post ultrasound volumes) and the measured actual voided volume using paired t test for the entire trial population and separately for within trial arms and; b) the accuracy of ultrasound (US) over measured actual voided volume with the use of the ratio of ultrasound derived voided volume to measured actual voided volume (Supplementary Table 1). US derived voided volume vs. measured volume were, mean ± standard deviation 364 ± 178 vs. 356 ± 185 ml *P* = 0.151 (entire trial population), 406 ± 138 vs. 390 ± 143 ml *P* = 0.058 (mobilize to toilet) and 322 ± 202 vs. 321 ± 215 ml *P* = 0.946 (bedpan); no significant difference between ultrasound derived to measured voided volumes in all three analyses indicating that ultrasound derived parameters are accurate and closely comparable to measured volumes. With accuracy defined as concordance within 20% range of ultrasound to measured actual voided volume in participants who managed to void (any volume, excluding complete overt retention), the accuracy rate by ultrasound 42/56 (75%) vs. 37/48 (77%) RR 95%CI 0.89 (0.36–2.20) *P* = 0.805 for toilet and bedpan arms respectively with no indication of operator bias across trial arms.

## Discussion

Nulliparas in labor randomized to mobilizing to the toilet were more likely to micturate satisfactorily (NNT_b_ 4.8) and less likely to have overt urinary retention (NNT_b_ 8.3). Amongst women who had overt urinary retention or complete failure to void with their allocated intervention, 13/15 (87%) who cross-over to the toilet after failure with the bedpan managed to micturate satisfactorily whilst no participant 0/3 (0%) who cross-over to the bedpan after failure in the toilet managed to micturate satisfactorily. However, after cross-over was permitted, the need for catheterization was low (toilet 5% vs. bedpan 4%) and not significantly different across trial arms based on original intention-to-treat analysis.

A PubMed search (https://pubmed.ncbi.nlm.nih.gov/) was performed on April 6 2021 using search terms “toilet bedpan labor trial” retrieved only a solitary trial report [21]. In that report, parturients with epidural analgesia for labor who managed the walk to the toilet, more managed to void, fewer required urinary bladder catheterization in labor and their post-void residual volume was significantly lower. However, 59% of the walk to toilet arm were unable to walk and actually voided in a bedpan [21]. These findings [21] concur with our findings of a better micturition performance in the privacy of the toilet amongst nulliparas in labor without neuraxial analgesia.

Participants in mobilization to the toilet arm were more likely to report satisfaction (NNT_b_ 1.3) and 95% would prefer to mobilize to the toilet to urinate if they needed to do so again. In women randomized to bedpan, although 74% achieved satisfactory micturition with bedpan, 91% would still prefer to mobilize to toilet to micturate, which suggested that the preference for the toilet was not motivated by a bad micturition performance with bedpan, but a positive personal choice and a near universal one at that. The bedpan is a subject of everyday nursing practice but little research was found concerning this issue; its use has been described by patients as uncomfortable, embarrassing [22, 23] and creating a feeling of dependency [23] which might have contributed to our satisfaction and preference findings in the disfavor of the bedpan.

Our primary outcome was predicated on ultrasound derived post-void residual volume which was calculated using the prolate ellipsoid method [15] We used the proportionality constant of 0.52 in our volume formula as it correlated best [15, 16] with measured volumes. Our post hoc analyses also showed that our ultrasound derived voided volumes were very similar to the measured actual voided volumes. There was also no indication of an operator bias in ultrasound measurements across trial arms (Supplementary Table 1).

The overall urinary catheterization rate in our trial was low at about 5% and similar across trial arms due to an almost 90% cross-over success rate after unsatisfactory micturition with the bedpan, whereas after unsatisfactory micturition in the toilet, cross-over success rate was 0%. This finding reinforced our primary finding of the toilet being the better set-up for micturition. Our data indicated that a two-step approach of bedpan and if unsatisfactory, switch-over to toilet compared to mobilization to the toilet might still be viable but cumbersome option with the catheterization rate as the endpoint. Use of the bedpan can be convenient but the privacy of the toilet appeared the most suitable for micturition.

With regard to strength, this is an original and powered study to evaluate the micturition performance in toilet or by bedpan of nulliparas in labor without neuraxial analgesia with positive finding on the primary and a number of supportive secondary outcomes in favor of the toilet environment. The study was powered and data replete with no drop-out. A single investigator was involved, eliminating inter-operator bias of ultrasound assessment. The investigator had 6 years of experience after certified training in obstetrics and gynecologic ultrasound. We believed our findings to be generalizable.

With regards to limitations, this study was not blinded to the investigator-sonographer. This could be important as our primary outcome was ultrasound derived post-void residual urine volume and there could be investigator bias in image capture and measurements. Post hoc analyses of our data on ultrasound accuracy did not suggest operator bias across trial arms. There is continuing debate about the accuracy of ultrasound estimation of urine volume particularly during labor compared to measured catheterized volumes and 2-dimensional vs. 3-dimensional bladder volumes using ultrasound [14, 16, 24–26]. In the bedpan arm, we allowed choice of lying down or sat up, and as posture could plausibly affect micturition performance, these choices could cause confounding. It is possible that women who are in the most advance stages of labor or very distressed are less likely to be recruited which may introduce selection bias.

## Conclusions

In conclusion, nulliparas in labor are more likely to have satisfactory micturition and far higher maternal satisfaction when mobilized to the toilet compared to bedpan use. However, with cross-over to go to the toilet if bedpan use is unsatisfactory, urinary catheterization rates are similar indicating a two-step approach with initial bedpan may still be viable. As a woman-centered quality of care issue, mobilizing to the toilet in labor to micturate is overwhelmingly preferred over the alternatives of the bedpan or catheterization.

## Supplementary Information

**Additional file 1.** 

## Data Availability

The datasets used and/or analyzed during the current study are available from the corresponding author on reasonable request.
