# Supplementary material for: Micturition in the toilet compared with bedpan in laboring Nulliparas: a randomized controlled trial
Source: BMC Pregnancy Childbirth. 2022 Nov 4;22:812. doi: 10.1186/s12884-022-05162-4 (PMC9635173; doi:10.1186/s12884-022-05162-4)
Supplement: Supplementary file 1 — Additional file 1. [file 12884_2022_5162_MOESM1_ESM.docx]

Supplementary Table 1. Post hoc analysis on ultrasound (US) derived vs. measured voided volume.

1. Entire trial population (N = 116)

|  | Voided volume | P value |
| --- | --- | --- |
| US derived voided volume (ml)  vs | 364 ± 178 | 0.151 |
| Measured voided volume (ml) | 356 ± 185 |  |
| Mean difference | 8.4 ± 63.1 |  |

1. Mobilizing to toilet arm (n = 58)

|  | Voided volume | P value |
| --- | --- | --- |
| US derived voided volume (ml)  vs | 406 ± 138 | 0.058 |
| Measured voided volume (ml) | 390 ± 143 |  |
| Mean difference | 16.4 ±64.7 |  |

1. Bedpan arm (n = 8)

|  | Voided volume | P value |
| --- | --- | --- |
| US derived voided volume (ml)  vs | 322 ± 202 | 0.946 |
| Measured voided volume (ml) | 321 ± 215 |  |
| Mean difference | 0.6 ±61.1 |  |

Data expressed as mean ± standard deviation. Analysis by paired t test.

1. Ratio of Ultrasound (US) over measured voided volume in participants who managed to void after allocated intervention.

| Outcomes | Mobilizing to toilet (n=56) | Bedpan  (n=48) | RR (95%CI) | P value |
| --- | --- | --- | --- | --- |
| Ratio of US voided volume to measured voided volume |  |  |  | 0.600 |
| Slight diferrence^1^ | 19 (34) | 11 (23) |  |  |
| Moderate underestimation^1^ | 9 (16) | 10 (21) |  |  |
| Significant underestimation^1^ | 3 (5) | 4 (8) |  |  |
| Moderate overestimation^1^ | 14 (25) | 16 (33) |  |  |
| Significant overestimation^1^ | 11 (20) | 7 (15) |  |  |
| Accuracy |  |  | 0.89 (0.36-2.20) | 0.805 |
| Accurate^2^ | 42 (75) | 37 (77) |  |  |
| Significantly difference^2^ | 14 (25) | 11 (23) |  |  |

Data expressed as number (%). Analyses by Chi square test.

^1^Ratio of ultrasound derived to measured voided volume classified as slight difference when 0.9-1.1 (± 10%), moderate underestimation and moderate overestimation when 0.8-0.9 and 1.1-1.2 (i.e., ± 10-20%), significant underestimation and significant overestimation when < 0.800 and > 1.2 (i.e., > ± 20%)

^2^Slight and moderate over/underestimation recategorize into accurate and significant under/overestimation to significantly difference.
